# Cyclophilin J Is a Novel Peptidyl-Prolyl Isomerase and Target for Repressing the Growth of Hepatocellular Carcinoma

**DOI:** 10.1371/journal.pone.0127668

**Published:** 2015-05-28

**Authors:** Jian Chen, Shuai Chen, Jiahui Wang, Mingjun Zhang, Zhaohua Gong, Youheng Wei, Li Li, Yuanyuan Zhang, Xuemei Zhao, Songmin Jiang, Long Yu

**Affiliations:** 1 State Key Laboratory of Genetic Engineering, Institute of Genetics, School of Life Sciences, Fudan University, Shanghai, P.R. China; 2 Shandong Research Center of Stem Cell Engineering, Yantai Yuhuangding Hospital, Affiliated Hospital of Qingdao University, Yantai, Shandong, P.R. China; 3 Department of Oncology, Yantai Yuhuangding Hospital, Affiliated Hospital of Qingdao University, Yantai, Shandong, P.R. China; University of Navarra School of Medicine and Center for Applied Medical Research (CIMA), SPAIN

## Abstract

Cyclophilin J (CYPJ) is a new member of the peptidyl-prolyl *cis/trans*-isomerase (PPIase) identified with upregulated expression in human glioma. However, the biological function of CYPJ remained unclear. We aimed to study the role of CYPJ in hepatocellular carcinoma (HCC) carcinogenesis and its therapeutic potential. We determined the expression of CYPJ in HCC/adjacent normal tissues using Western blot, Northern blot and semi-quantitative RT-PCR, analyzed the biochemical characteristics of CYPJ, and resolved the 3D-structure of CYPJ/Cyclosporin A (CsA) complex. We also studied the roles of CYPJ in cell cycle, cyclin D1 regulation, *in vitro* and *in vivo* tumor growth. We found that CYPJ expression was upregulated in over 60% HCC tissues. The PPIase activity of CYPJ could be inhibited by the widely used immunosuppressive drug CsA. CYPJ was found expressed in the whole cell of HCC with preferential location at the cell nucleus. CYPJ promoted the transition of cells from G1 phase to S phase in a PPIase-dependent manner by activating cyclin D1 promoter. CYPJ overexpression accelerated liver cell growth *in vitro* (cell growth assay, colony formation) and *in vivo* (xenograft tumor formation). Inhibition of CYPJ by its inhibitor CsA or CYPJ-specific RNAi diminished the growth of liver cancer cells *in vitro* and *in vivo*. In conclusion, CYPJ could facilitate HCC growth by promoting cell cycle transition from G1 to S phase through the upregulation of cyclin D1. Suppression of CYPJ could repress the growth of HCC, which makes CYPJ a potential target for the development of new strategies to treat this malignancy.

## Introduction

Hepatocellular carcinoma (HCC) is one of the most common cancers worldwide and a leading cause of death in many countries, particularly in East Asia and Africa. The onset and progression of HCC is related to risk factors such as chronic infection with hepatitis B and C viruses and exposure to hepatocarcinogen aflatoxin B1 [1]. The development of HCC is a multi-step process associated with aberrations in chromosomes and alteration in gene-expression patterns. Many efforts have been made to identify potential tumor suppressor genes in these as well as other regions of the genome, culminating in the discovery of inactivating point mutations in a number of tumor-suppressor genes such as p53, p16, p21, beta-catenin, *PTEN*, and Rb [2–6]. Despite the completion of the Human Genome Project and the sequencing of increasing number of defined cell lines [7], it remains a challenge to identify novel HCC related genes important for the disease.

Cyclophilins constitute a superfamily of peptide-prolyl isomerases (PPIase), which catalyzes the *cis-trans* isomerization of peptide bonds on the NH-terminal side of Pro residues [8]. Cyclophilins have been shown to act as chaperons to accelerate protein folding and maturation and play critical roles in signal transduction [9]. The cyclophilin family is comprised of more than fifteen members and was named for their ability to bind the widely used immunosuppressive drug cyclosporine A (CsA) [10]. Cyclophilins have been implicated in many pathological processes, including virus infection [11], rheumatoid arthritis [12], cardiovascular diseases [13] and cancer [14,15]. The precise role of cyclophilins in promoting tumorgenesis, however, has remained largely unknown.

To identify genes involved in the development of HCC, we previously carried out digital differential analyses by comparing the expression of ESTs (expressed sequence tags) in human HCC and normal liver tissues. Among several differentially expressed ESTs, one cDNA upregulated in HCC with a high degree of sequence similarity to human cyclophilin A was chosen for further characterization (unpublished data). The full-length cDNA was cloned and sequenced. It was found to be the new member of the cyclophilin superfamily and was thus named Cyclophilin J (CYPJ, Genbank association number AF146799). Cyclophilin J has also been cloned by another laboratory under the name of *PPIL3* (Peptide-Prolyl Isomerase-Like 3) [16], and its upregulation in human glioma was reported [17]. However, the biological function of CYPJ remained unclear. Here, we report a frequent upregulation of *CYPJ* in HCC which promotes the growth of liver cells. In addition, the inhibition of CYPJ leads to suppression of HCC growth. Our findings are important for a better understanding of the molecular mechanisms underlying the tumorgenesis of HCC, and suggest that CYPJ may serve as a novel therapeutic target for HCC.

## Materials and Methods

### Cloning of cDNA for CYPJ

The full-length nucleotide sequence of human cyclophilin J was predicted based on its EST sequence and its cDNA was cloned from human multi-tissue cDNA libraries (Clontech, Inc.) by RT-PCR (forward primer: 5’-AAGACTGAGAAATCACGTAGTCC-3’; reverse primer: 5’-CAAGCAGAAGGATGATGCAATC-3’).

### Samples of primary HCC, adjacent tissues, and cell culture

All samples of primary HCC (T) and adjacent non-tumorous tissues (N) were obtained from Department of Oncology of Yantai Yuhuangding Hospital (Yantai, China). No patient received radiotherapy or chemotherapy before sampling. Most patients with HCC (94.6%) were positive for HBV surface antigen. Fetal liver tissues were obtained from the Gynecology Department of Yantai Yuhuangding Hospital (Yantai, China). All tissues were placed in liquid nitrogen immediately after surgical resection. Hep3B, HepG2, Hela, COS7, and HEK-293T cells were cultured at 37°C with 5% CO_2_ in Dulbecco’s Modified Eagle Medium (DMEM; Gibco-BRL Inc.) supplemented with 10% fetal calf serum (FCS; Gibco-BRL Inc.), and YY8103, L02, and SK-Hep1 cells were cultured in RPMI-1640 Medium (Gibco-BRL Inc.) supplemented with 10% FCS.

### Northern blot

Total RNA was extracted with Trizol reagent (Invitrogen) in accordance with the manufacturer’s protocol. The gene-specific PCR fragments of CYPJ cDNA was labeled with α-^32^P-dATP with random primer kit (Amershan) to hybridize MTN membranes carrying mRNA from 16 human tissues (Clontech) or nylon membranes carrying total RNA from resected liver specimen of 16 cases of HCC and 2 fetal livers. The membranes were prehybridized in Hybridization/Prehybridization solution (50% formamide, 5 × SSPE, 10 × Denhardt’s solution, 2% SDS, 100 mg/l calf-thymus DNA) at 42°C for 24 h, followed by hybridizing with labeled probe for additional 24 h. The membranes were washed for three times in wash solution (2 × SSC/0.1% SDS; 0.5 × SSC/0.1% SDS; 0.1 × SSC/0.1% SDS) at 65°C before exposure to X-ray film at -80°C for 5 days. As a control, MTN I and MTN II were also hybridized with a 2.0 kb β-actin (*ACTB*) cDNA under the same condition, followed by a 4 h exposure to X-film at -80°C. For other membranes, the results of total RNA electrophoresis were used as controls.

### Semi-quantitative RT-PCR

cDNA was synthesized using 2 mg of total RNA, SuperscriptII reverse transcriptase (Gibco-BRL Inc.) and Oligo(dT)15 (Promega) according to the manufacturer’s protocols. First-strand cDNA was subjected to RT-PCR amplification on FS-918 DNA Amplifier (Shanghai Fusheng Institute of Biotechnology). To optimize the cycle number, PCR amplifications were performed for 20–37 cycles (94°C 30 s, 60°C 30 s, 72°C 30 s). The products from each cycle were separated and visualized on a 2% agarose gel upon electrophoresis and the growth curve of the PCR products was made according to the amount of PCR products in different cycles to determine the optional cycle number. The semi-quantitative RT-PCR results from 40 HCC samples were scanned with GDS-800 (Bio-Rad) and Annutating Grabber 1T2.51 Scanner software as well as UVP Gelworks ID Advanced Version 2.51 analysis software. The *CYPJ* mRNA levels in cancer and normal tissues were calculated using a dosage ratio (DR) of the ethidium bromide intensity of *CYPJ*/*ACTB* bands in agarose gels [18].

### Subcloning

For prokaryotic expression, The ORF of *CYPJ* was amplified with forward primer 5’-ATAAGAATGCGGCCGCTCTGTGACACTGCATA-3’ and reverse primer 5’-ATCGCT CGAGCTGAGCAAATGGGTTGGCAT-3’ which contained the NotI and XhoI restriction sites respectively, to allow directional subcloning into the pTXB1 vector (New England Biolab). For eukaryotic expression, the coding sequences of *CYPJ* and *CYPA* were subcloned in-frame into the pCMV-HA vector (Clontech). The catalytic mutants of CYPJ (R44A, R44A&F49A, and K120A) and CYPA (R55A&F60A) were generated using QuikChange Site-Directed Mutagenesis Kit (STRATAGENE) in both systems. For stable cell line generation, the coding sequence of CYPJ was subcloned in-frame into the pcDNA3.1-myc vector (Clontech).

### Expression and purification of recombinant human CYPJ protein

Expression plasmids for CYPJ and the three mutants were transformed into *E*. *coli* strain ER2566 (New England Biolab). Recombinant proteins were expressed following a 20 h induction with 0.2 mM IPTG at 22°C, and were subsequently purified by Chintin Beads system following the manufacturer’s protocol. Briefly, crude extracts from *E*.*coli* containing fusion protein were passed over a 1 ml column at 4°C. The column was washed with >10 column volumes of washing buffer (20 mM HEPES, pH 8.0, 500 mM NaCl, 0.1 mM EDTA, and 0.1% Triton-X100). The column was then quickly flushed with 3 column volumes of fresh cleavage buffer (20 mM HEPES pH 8.0, 50 mM NaCl, 0.1 mM EDTA, and 30 mM DTT). The flow to the column was stopped, and the column was placed at 4°C overnight. CYPJ was eluted using 3 column volumes of cleavage buffer without DTT. Purified protein was analyzed by LC/MS, or kept in PPIase buffer (50 mM HEPES, pH 8.0, and 86 mM NaCl) at 4°C until use.

### PPIase assay

The assay for PPIase activity of CYPJ and its mutants was modified from the methods of Kofron [19]. The experiment was performed under 8.5°C in a 100 μl system as following: A sample of 2 μl (65.6 μmol/l) of protein was added to 86 μl of PPIase buffer and incubated on ice for 1 h. Reaction was started by the addition of 10 μl chymotrypsin (60 mg/ml solution) and 2 μl peptide substrate N-succinyl-Ala-Ala-Pro-Phe-p-nitroaniline (Sigma) at different concentrations followed by rapid mixing. The absorbance change at 390 nm due to the release of *p-*nitroaniline was recorded using U-3000 spectrophotometer (Hitachi). CsA inhibition assays were carried out as described above except that the samples were preincubated for 60 min with varying amounts of CsA.

### Crystal structure analysis

The structure of human CYPJ refined at 2.6 Å resolution [20] was used as a starting model, in which the solvent molecules were omitted, and the structure was further refined using X-ray data collected at 2.0 Å resolution on an R-AXIS IV++ detector [21]. The data were rescaled using HKL2000 software [22], leading to space group P3121 with 11753 unique reflections. Crystallographic refinement was carried out using program CNS [23], and manual refitting of the CYPJ model was performed using program TURBO-FRODO [24]. Solvent molecules were added to the model at late stage of the refinement and only those with B values lower than 50 Å2 were included in the final structure.

Single crystals of CYPJ-CsA complex were grown using the hanging drop vapor diffusion method at 293 K under the following conditions: protein solution containing 0.5 mM CYPJ, 1mM CsA, 4% DMSO and 4% isopropanol was mixed at a ratio of 1:1, with the reservoir solution containing 0.1 M Tris-HCl (pH 7.4), 18% PEG 8000 (*w/v*) and 6% DMSO. Two sets of X-ray data were collected at 293 K with CCD detectors, using a laboratory X-ray source at 2.7 Å resolution for data set 1 and later using Beijing Synchrotron Radiation Facility at 2.4 Å resolution for data set 2, respectively. Both sets of data were processed using software HKL2000. The three-dimensional structure of CYPJ-CsA complex was determined by molecular replacement using program AMoRe [25], and the molecular structure of CYPJ at 2.6 Å resolution was used as the search model [20]. The structure was refined using data sets 1 and 2 successively, in a procedure similar to that used to refine CYPJ. The crystal data and data collection statistics of data collection and crystallographic refinement are shown in S1 Table.

### Eukaryotic expression of CYPJ and selection of stably transfected cells

The eukaryotic expression plasmids of CYPJ, CYPA and their mutants were transfected into cells using Lipofectamine 2000 reagent (Invitrogen) and the expressed proteins were detected using western blot. For stable cell line generation, HCC cell line SK-Hep1 and liver cell line L02 were transfected with pcDNA3.1-CYPJ or control plasmid pcDNA3.1-myc with Lipofectamine 2000 reagents. Sixty hours after transfection, G418 (Life Technologies Inc.) was added to the medium at a final concentration of 600 μg/ml. After selection for 3 weeks, clones were selected and expanded. The expression of myc-tagged CYPJ in each individual clone was confirmed by western blot.

### Western blot

Samples were separated by 10% SDS-polyacrylamide gel electrophoresis, followed by transfer to PVDF membrane. After blocking in phosphate-buffered saline containing 5% bovine serum albumin and 0.1% Tween-20, the membrane was incubated with anti-myc monoclonal antibody (Cell Signaling Technology Inc.), anti-HA monoclonal antibody (Cell Signaling Technology Inc.), anti-Cyclin D1 monoclonal antibody (Santa Cruz Inc.), anti-PPIL3 polyclonal antibody (Abcam) or anti-β-actin monoclonal antibody (Sigma) at room temperature for 2 h, followed by incubation with a peroxidase-linked secondary antibody (CalbioChem) at room temperature for one h. The signals were detected using Western blotting Luminol Reagent (Santa Cruz Inc.).

### Subcellular location, cell cycle, and cell growth analyses

#### Subcellular location analysis

The coding sequence of *CYPJ* was inserted in frame into pEGFP-N1 (Clontech). The resultant pEGFP-CYPJ fusion protein-expression plasmid was transfected to Hela cells and visualized using methods described previously [26]. DAPI staining was used to locate the nucleus of Hela cells. In a separate experiment, pCMV-CYPJ plasmid was transfected into Hela cells, and 40 h after transfection, the cells were fixed in 4% paraformaldehyde. The subcellular location of CYPJ was detected by anti-HA mAb and subsequently visualized by Rhodamine-conjugated secondary antibody.

#### Cell cycle analysis

Cells were harvested and fixed in 70% ethanol at -20°C for 24 h before they were resuspended with 10 μl propidium iodide (Life Technologies Inc.) followed by incubation at room temperature for 20 min. Cells were analyzed on a FACSVantage SE instrument (BD Biosciences Pharmingen, San Diego, CA). The experiments were repeated three times.

#### Colony formation assay

L02 cells (approximately 2×10^5^ cells per 60-mm dish) were transfected with 2 μg of pcDNA3.1-CYPJ or empty vector pcDNA3.1-myc with Lipofect2000 reagent. Twenty-four hours after transfection, G418 was added to the medium at a final concentration of 600 μg/ml. Cells were under G418 selection for 3 weeks. Colonies were fixed with methanol and stained with Giemsa to enumerate the transformed foci.

#### Cell growth analysis

Ninety-six-well plates were seeded for triplicate with SK-Hep1-CYPJ cell clones and SK-Hep1-pcDNA control clones at 1×10^3^ cells/well. From the first day to the fifth day, cell growth was monitored by absorbance using MTS assay (Promega Inc.). To examine the inhibitory effects of CsA on the growth of several liver cancer cells, 96-well plates were seeded with Hep3B, HepG2, YY8103, QGY, SK-Hep1 and SK-Hep1-CYPJ cells at 1 × 10^3^ cells/well. After the treatment with different concentrations of compounds for 72 h, cell viability was monitored by MTS assay compared with control cells treated with PBS.

### Promoter reporter assay for cyclin D1

The human cyclin D1 promoter-luciferase reporter plasmids -962CD1 and its deletion mutants constructed in pGL3-basic vector were generously provide by Drs. O. Tetsu and F. McCormick (UCSF, San Francisco, CA) [27]. The reporter construct (100 ng/well in 24-well plates) was cotransfected into HEK-293T cells with pCMV-CYPA, pCMV-CYPA(R55A&F60A), pCMV-CYPJ, pCMV-CYPJ(R44A&F49A), or the control pCMV-HA vector alone (200 ng/well for each vector). Thirty hours after transfection, cell lysates were prepared and luciferase activity was determined using Luciferase Assay System. Plasmid pRL-SV40 (Promega; 30 ng/well) encoding Renilla luciferase was used as an internal control in each transfection. To test the inhibitory effect of CsA on CYPJ-mediated activation of cyclin D1 promoter, CsA was added to the media at different concentrations 12 h after the co-transfection assay described above.

### Quantitative real-time PCR

Quantification of mRNA was performed by SYBR Green Ⅰ staining (Takara, Inc.) on an iCycler iQ system (BioRad). To compare the relative levels of gene expression, we used cDNA from a normal adult brain (Clontech, Inc.) to generate standard amplification curve. The primers for amplification are listed in S2 Table. The optimal conditions for PCR were as follows: 40 cycles of three-step PCR (95°C for 50 s, 63°C for 1 min, and 72°C for 30 s) after initial denaturation (95°C for 5 min). The real-time PCR reactions were performed in 25 μl volumes according to the manufacturer’s protocol. The relative mRNA levels of each gene in different cDNA sample were normalized to the internal levels of *GAPDH* individually. Experiments were carried out twice for each data point, and negative controls were performed to avoid genomic DNA contamination.

### RNAi

siRNA duplexes (si-CYPJ, 5'-CTGGAAGAGGAGGCAACAG-3') containing 3'dTdT over-hanging sequences were synthesized at Shanghai GeneChem Co. A control nucleotide (si-control) was also purchased from GeneChem. The sequences of si-CYPJ did not have significant homology to mRNA for other CYPs. The siRNA duplexes were co-transfect with pCMV-CYPJ plasmids and its efficiency to knockdown exogenous CYPJ expression was examined by western blot. Knockdown of endogenous *CYPJ* mRNA in SK-Hep1 cells was verified by RT-PCR and quantitative real-time RT-PCR. Lentivirus containing sh-RNA for CYPJ kockdown was also generated at Shanghai GeneChem Co., Ltd (sense sequence: gCTGGAAGAGGAGGCAACAG; antisense sequence: CTGTTGCCTCCTCTTCCAGc; loop sequence: TTCAAGAGA).

### Tumor development in athymic nude mice

Male nude mice (BALB/c-nude, 4–5 week old) were inoculated subcutaneously with CYPJ transfectant and vector control cells (2.5×10^6^ cells suspended in 0.2 ml of PBS) and monitored for tumor development. Mice were sacrificed and tumors were removed 40 days after inoculation.

### *In vivo* gene transfer in subcutaneous tumor model

HCC cells SK-Hep1 (6 × 10^6^ cells suspended in 0.2 ml of PBS) were implanted into the left flanks of BALB/c-nude mice, and 7 days later, when tumors reached 3–4 mm in diameters, an intra-tumor injection of LV-CYPJ-RNAi and LV-non-silencing control at a titer of 3 × 10^7^ TU in 20 μl PBS was administrated, and the same treatment was repeated two days later. Mice were monitored daily and tumor sizes were measured every 3 days. Mice were sacrificed 23 days after the final injection and the tumors were removed for analyses [28].

### Immunohistochemical staining

The harvested tissue was sliced into 4 micrometers sections. The sections were deparaffined in xylene and rehydrated in alcohol. Endogenous peroxidase was perished by 3% H_2_O_2_ for 10 min. Antigen retrieval was achieved by treatment of microwave in citrate acid buffer (pH 6.0) twice, and then blocked in 3% goat serum (Boster China) for 15 min in 37°C for any none specific reaction, incubated in primary antibody in 4°C overnight, and washed in PBS for three times. The slides were further incubated with biotin labeled secondary antibody for 30 min, washed three times in PBS, incubated in SABC complex for 25 min, washed again in PBS for three times, and visualized using 3,3’-diaminobenzidine tetra hydrochloride (DAB/H_2_O_2_) and counterstained with hemotoxylin. In lentivirus-mediated gene knockdown experiments, a DAPI staining was used to detect the nucleus of tumor cells, and the green fluorescence (GFP carried by lentivirus) was used to indicate transfection efficiency.

### Statistics

Statistical difference was analyzed using student’s T-test where *P*<0.05 suggested statistically significant difference.

### Ethics Statement

This study involved using of human tissues, in which written informed consents from the donors or their next of kin were obtained for the use of these samples in research. All clinical investigations have been conducted according to the principles expressed in the Declaration of Helsinki. The use of human tissues and animal experiments were approved by the Ethics Committee of Yantai Yuhuangding Hospital, China. Mice were sacrificed by cervical dislocation performed by an individual with a demonstrated high degree of technical proficiency

## Results

### Elevated CYPJ expression in hepatocellular carcinoma

**Fig 1:**
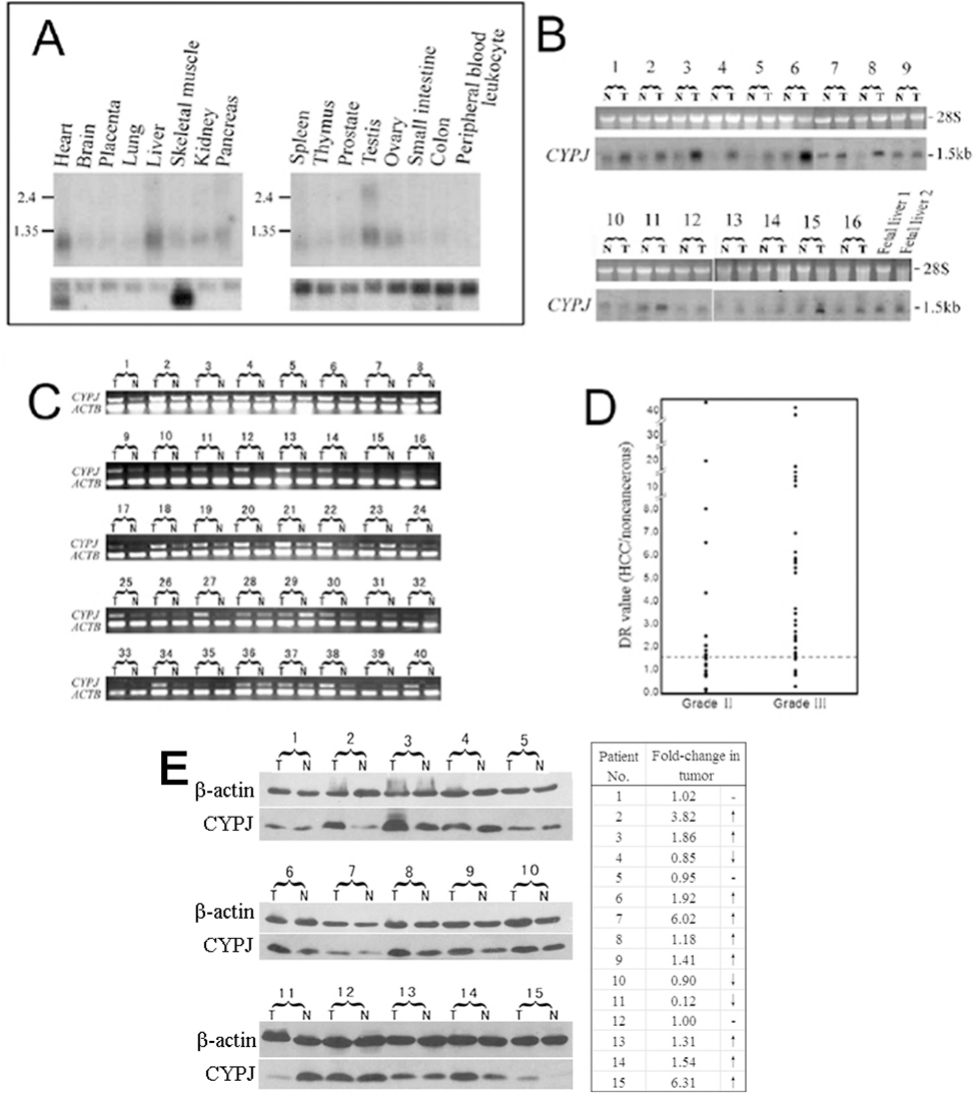
Expression pattern of *CYPJ* gene and its upregulation in HCC. (A) Northern blot analysis of *CYPJ* in 16 main human tissues. (B) Northern blot analysis of *CYPJ* expression in 16 paired HCC/adjacent liver tissues and two fetal liver tissues. (C) Semi-quantitative RT-PCR analysis of *CYPJ* expression in 40 paired HCC/adjacent tissues. (D) Stage plots of deregulated CYPJ levels in 56 paired HCC/adjacent liver tissues. *CYPJ* levels were determined by Northern blot/RT-PCR. β-actin was used as internal control, and DR values were calculated. (E) Western blot of CYPJ expression in 15 paired HCC/adjacent normal tissues using anti-PPIL3 antibody. β-actin was used as internal control. Fold change of CYPJ expression in tumor = (T/T_C_)/(N/N_C_). T: tumor tissue sample. N: adjacent normal tissue sample. T_C_: tumor sample control. N_C_: normal tissue sample control.

The tissue distribution of human CYPJ was determined by Northern hybridization with a α-^32^P dATP-labled gene-specific fragments as a probe (Fig 1A). A 1.3 kb transcript was detected in human adult tissues (MTN I and II, Clontech), with high levels of expression in the heart, liver, and testis, moderate levels of expression in skeletal muscle, kidney, pancreas, and spleen. *CYPJ* mRNA was barely detectable in the brain, placenta, lung, thymus, prostate, small intestine and colon, and no signal was detected in peripheral blood leukocyte. In addition to the 1.3 kb transcript, a 2.5 kb transcript could also be detected in the testis, which suggested the existence of another variant of human CYPJ specifically expressed in the testis.

The high level of human *CYPJ* in the liver and the possible link between cyclophilins and cancer cells prompted us to check its expression in HCCs, a malignancy with high incidence in China. The expression of human *CYPJ* mRNA was upregulated in the majority of (15 out of 16) tumorous specimens compared with adjacent normal tissues (Fig 1B; *P<*0.001). We also examined the expression of human *CYPJ* in fetal livers and found that *CYPJ* had higher expression in fetal livers than in normal adult livers (Fig 1B). Independently, we examined the expression of *CYPJ* in 40 pairs of HCC/adjacent non-cancerous tissues by semi-quantitative RT-PCR, and the results were in agreement with that from the Northern blot analyses, in which human *CYPJ* was found to be elevated in 26/40 (65%) tumor specimens (Fig 1C). The relationship of elevated CYPJ expression with clinical grades of HCC was also examined (Fig 1D). *CYPJ* was found to be upregulated in 12/22 (54.5%) HCC patients with Grade Ⅱ disease and in 28/34 (82.5%) HCC patients with Grade Ⅲ disease (*P*<0.01). In addition, the level of CYPJ in another 15 pairs of HCC/adjacent non-cancerous tissues samples was investigated by Western blot. Result showed that in 9/15 (60%) HCC samples an elevated CYPJ expression was observed (Fig 1E), which was consistent with the Northern result. In 3/15 (20%) samples the expression level of CYPJ remained unchanged while in 3/15 (20%) samples decreased. These data suggested that CYPJ was upregulated in HCC tissues which might play a role in the progression of the malignancy.

### CYPJ is a novel PPIase which binds to and is inhibited by CsA

**Fig 2:**
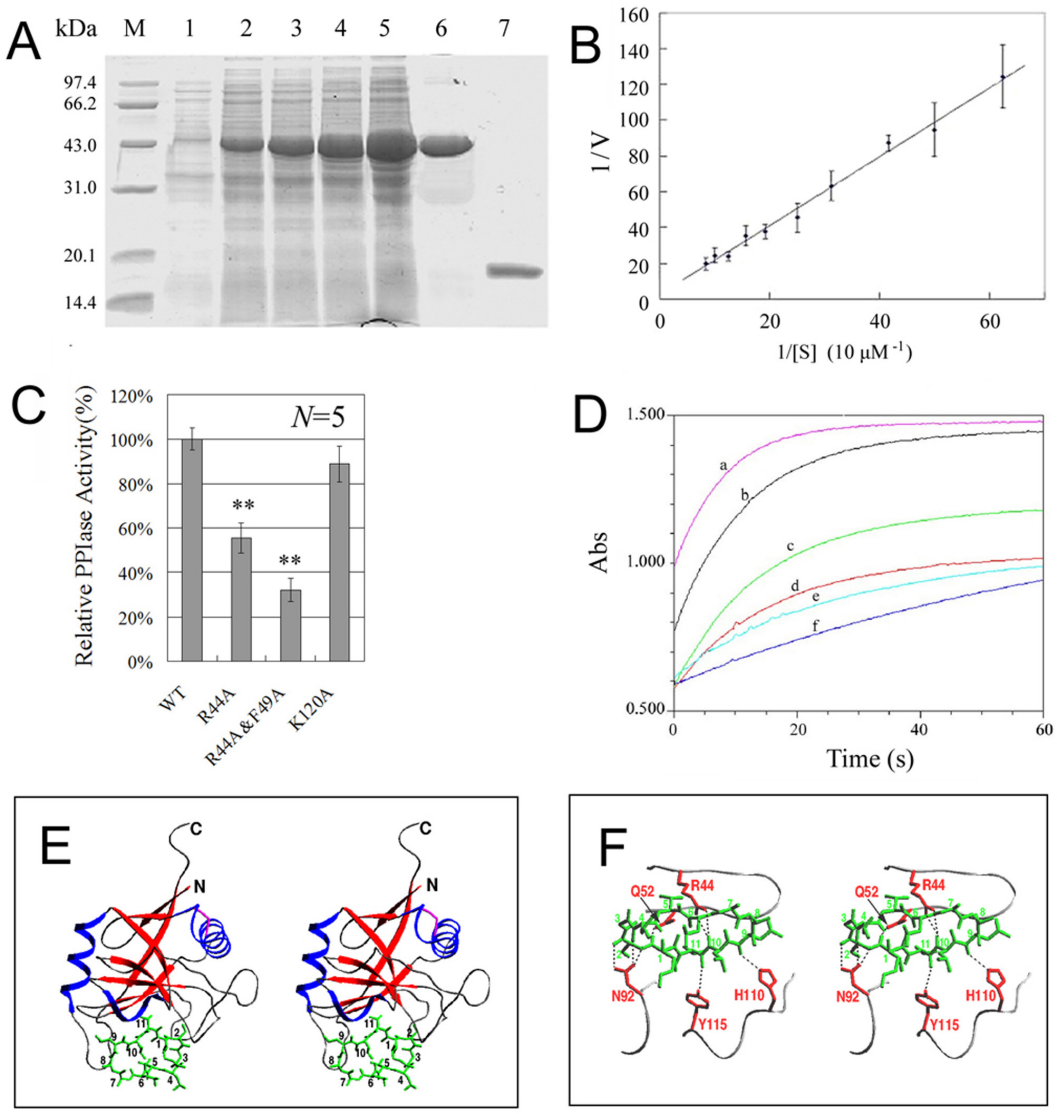
Biochemical characteristics of CYPJ protein and the 3D-structure of CYPJ/CsA complex. (A) Expression and purification of CYPJ. Line 1: un-induced *E*.*coli* lysate; lines 2–5: transformed *E*.*coli* was induced by 0.2 mM IPTG for 2, 3, 4 and 5 h, respectively; line 6: purified tagged CYPJ; line 7: the chitin tag was removed by DTT treatment. (B) Michaelis-Menten kinetics of CYPJ. (C) Effects of several site mutations (R44A, R44A&F49A, and K120A) to the peptidyl-prolyl *cis/trans*-isomerase activity of CYPJ. ** *P*<0.01. (D) Catalytic curves of inhibition. a, PPIase assay with 13.1 μM recombinant CYPJ protein; b-e, PPIase assays with 13.1 μM CYPJ and 1, 4, 8 or 16μM CsA, respectively; f, reference assay with no CYPJ or CsA. (D) and (E), 3D-structure of CYPJ-CsA complex. (D) CYPJ-CsA complex structure. CYPJ is shown as ribbon, and CsA is shown in green. The structure of CYPJ-CsA complex refined at 2.4 Å resolution contains two molecules of the complex in an asymmetric unit. In the final structure model the two CYPJ chains consist of 159 and 160 amino acid residues, respectively (the last two C-terminal residues in molecule A and the last one in molecule B could not be located). (E) A part of hydrogen bonding interactions between CYPJ and CsA. The side chains of Arg44, Gln52, Asn92, His110 and Tyr115 are shown in red and CsA in green. Hydrogen bonds are shown as dotted lines.

The deduced amino acid sequence of CYPJ showed 50% similarity with human CYPA and 72% similarity with CYP-10 of *C*. *elegant*. CYPJ also contained the PPIase consensus sequence [F/Y]–X(2)–[S/T/C/N/L/V]–X–F–H–[R/H]–[L/I/V/M/N]–[L/I/ V/M]–X(2)–F–[L/I/V/M]–X–Q–[A/G]–G [29]. These characteristics indicated that CYPJ may be a novel peptidyl-prolyl *cis/trans*-isomerase. To characterize CYPJ biochemically, we expressed and purified recombinant CYPJ in *E*. *coli* (Fig 2A). The relative molecular weight of the purified protein was 19379.34 Da as determined by LC/MS.

The enzymatic activity of human CYPJ was determined. In the chymotrypsin-coupled assay with different concentration of the peptide substrate, the recombinant human CYPJ appeared to follow Michaelis-Menten kinetics (Fig 2B). The value of k_cat_ and K_M_ were estimated to be 34.2±8.3 s^-1^ and 814±152 μM by a double reciprocal Lineweaver-Burke plot of 1/v against 1/[S], yielding a k_cat_/K_M_ value of (4.20±1.29)×10^4^ M^-1^S^-1^. Several key amino acids (R44, F49, K120) have been previously reported to be important for the catalytic activity of CYPA protein [30]. The corresponding mutants were generated in CYPJ (R44A, R44A&F49A, and K120A) and their residual PPIase activities were detected and compared with that of the wild-type CYPJ protein. The R44A, R44A&F49A, and K120A CYPJ mutants displayed 57%, 32% and 87% of the wild type enzyme activity respectively, consistent with the roles of these residues in catalysis (Fig 2C). In addition, the inhibitory effect of CsA on PPIase activity of CYPJ was also examined. CsA inhibited CYPJ in a dose-dependent manner (Fig 2D) with an IC_50_ of 12 μM in the current assay.

The 3D-structures of CYPJ and the CYPJ/CsA complexes were determined to explore the catalytic mechanism and key residues involved in CsA binding. The structure of CYPJ refined at 2.0 Å resolution showed similarity to that of CYPA (S1A Fig) as previously described [20]. His43, Arg44 and Gln52 were conserved active-site residues located in the shallow pocket, and Arg44 displayed flexible conformation as shown by comparison with CYPA and its various complexes (S1A and S1B Fig), suggesting that Arg44 is an important residue and its flexible conformation is essential for binding different substrates. This was consistent with the experimental data that mutation of Arg44 could significantly weaken its activity (Fig 2C). The structure of CsA in the CYPJ-CsA complex was similar to that in the CYPA-CsA complex structure (Fig 2E and 2F) [31]. However, the side chains of MeLeu4 and MeLeu6 displayed different conformations in the two structures, which belong to the effector domain (residues 4–8) of CsA and were involved in the interactions of CsA with calcineurin (Cn) [32]. On the other side of the cyclic undecapeptide, the cyclophilin-binding domain of CsA (residues 1–3, 9–11) interacted with CYPJ (Fig 2E). The hydrogen-bonding interactions between CYPJ and CsA were listed in S3 Table, including several C-H···O hydrogen bonds. Most of these interactions were conserved in CYPA-CsA complex structure (Fig 2F). Arg44 was involved in the hydrogen-bonding interactions with CsA, which well accounted for the experimentally observed inhibition of CsA on PPIase activity of CYPJ.

### Subcellular localization of CYPJ

**Fig 3:**
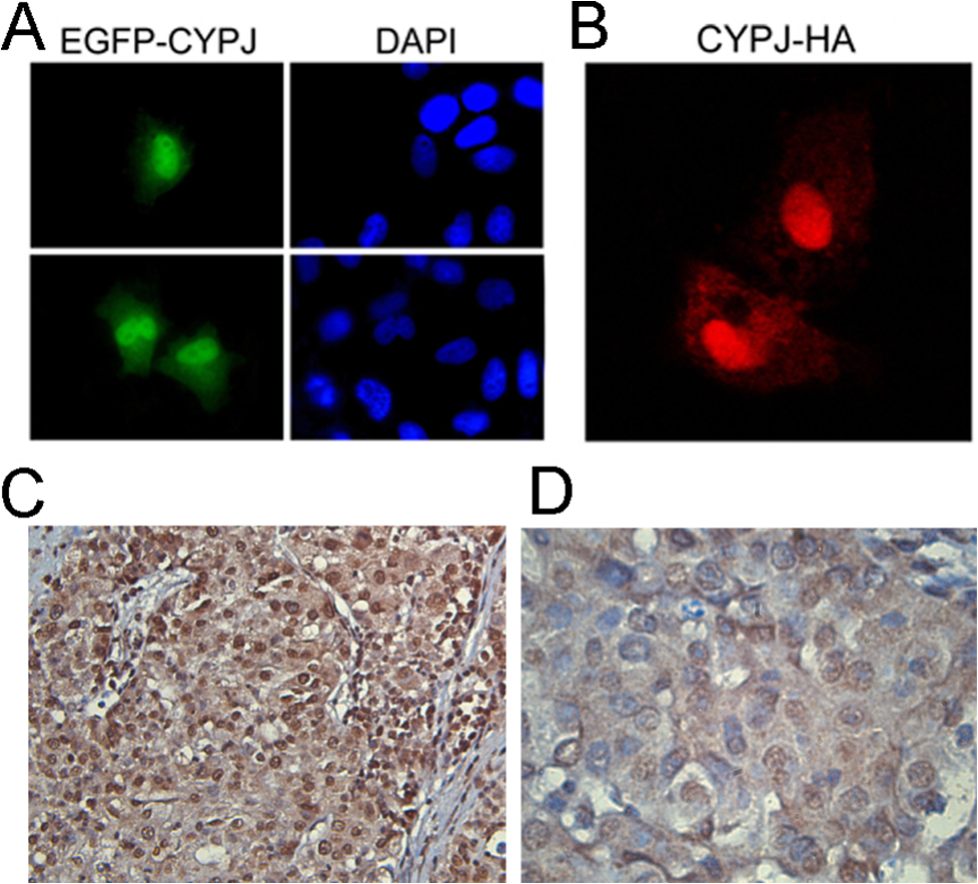
Subcellular localization of CYPJ. (A) Hela cells were transfected with pEGFP-CYPJ. After 48 h, cells were fixed and stained with DAPI to indicate the nuclei. (B) Hela cells were transfected with pCMV-CYPJ-HA, and 48 h later, the cells were fixed and stained with anti-HA monoclonal antibody. (C) and (D) Immunohistochemical stain of human HCC tissue section using anti-PPIL3 antibody. (C) ×100 magnification. (D) ×400 magnification.

Three methods were used to determine the subcellular localization of CYPJ: immunofluorescence microscopy for direct visualization of EGFP-fused CYPJ, indirect visualization for HA-tagged CYPJ in Hela cells, and immunohistochemical staining of HCC tissue sections. EGFP-fused CYPJ expression plasmid pEGFP-N1-CYPJ was transfected into Hela cells, and EGFP-CYPJ fusion protein was directly visualized by fluorescence microscope (Fig 3A). HA-tagged CYPJ-expressing plasmid pCMV-CYPJ was transfected into Hela cells, and the tagged CYPJ was detected by immunofluorescence (Fig 3B). CYPJ was found in both cytoplasm and the nucleus, with more distribution in the nucleus, which was also confirmed in HCC tissue by immunohistochemical analysis (Fig 3C and 3D). The preferential localization of CYPJ in the nucleus suggested that it might have a nuclear function.

### Involvement of CYPJ in the regulation of cell cycle

**Fig 4:**
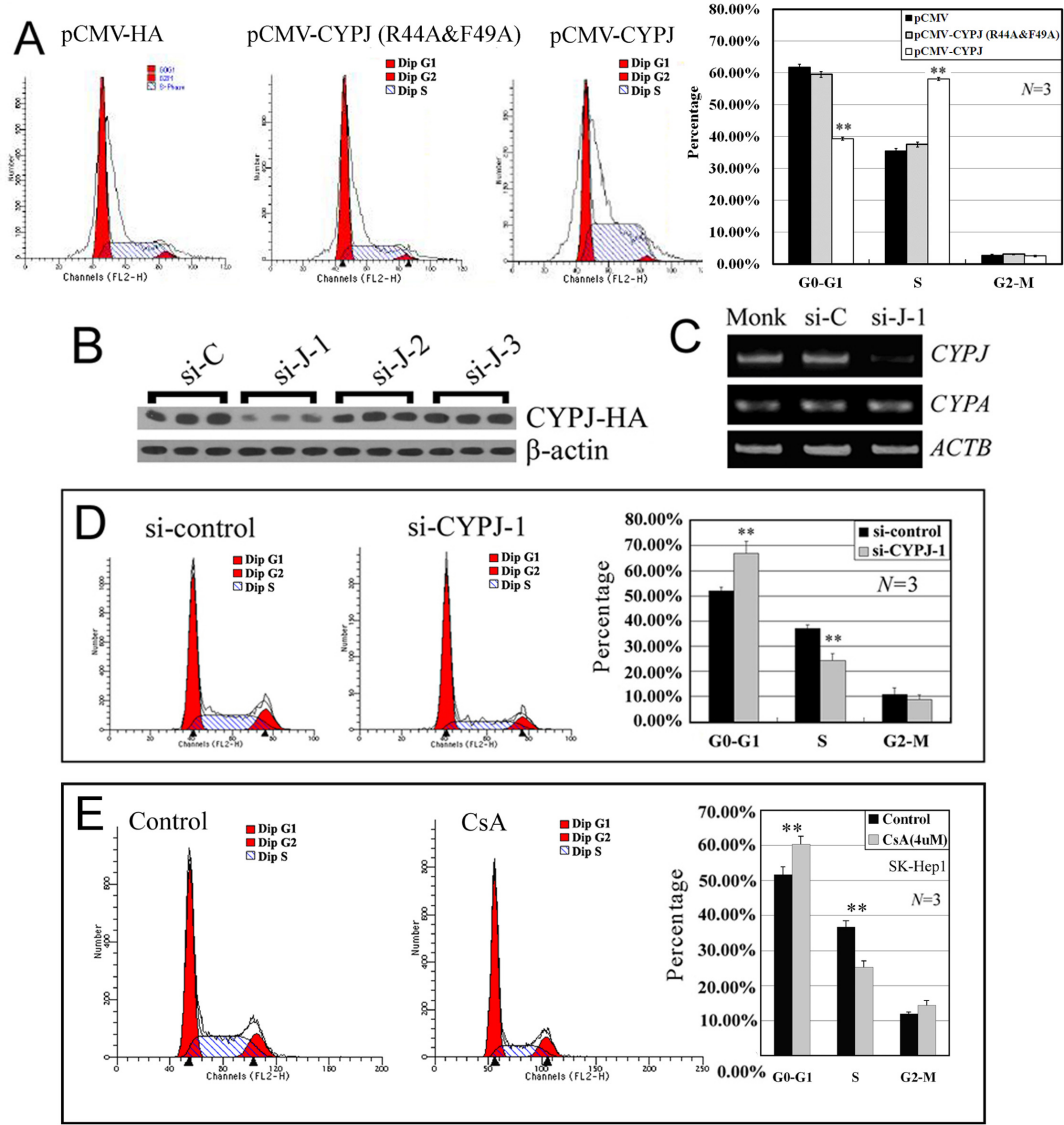
Effects of CYPJ on cell cycle distribution. (A) Effects of CYPJ and CYPJ(R44A&F49A) overexpression on cell cycle distribution of SK-Hep1 cells. ** *P*<0.01, N = 3. (B) Knockdown of exogenously enforced CYPJ expression by three different short interference RNAs. The most effective siRNA complex si-J-1 was selected for further analyses. (C) Knockdown of endogenous *CYPJ* expression by short interference RNA. The expression of *CYPA* was not affected by the si-J-1 transfection. β-actin was used as internal control. (D) Effects of CYPJ knockdown on cell cycle distribution of SK-Hep1 cells. ** *P*<0.01, N = 3. (E) Effects of CsA inhibition on cell cycle distribution of SK-Hep1 cells. Cells were treated with 4 μM CsA for 48 h before the experiment. ** *P*<0.01, N = 3.

To assess the potential role of CYPJ in the regulation of cell cycle, we examined and compared cell distribution of human hepatic cancer cell line SK-Hep1 cells transfected with empty vector pCMV-HA, cells overexpressing either CYPJ or PPIase-impaired CYPJ mutant from pCMV-CYPJ or pCMV-CYPJ(R44A&F49A) respectively, cells with CYPJ knockdown by CYPJ-siRNAs and inhibition of CYPJ PPIase activity by CsA. Compared with empty vector-transfected control cells, overexpression of CYPJ induced an increase in cell population in the S phase (from 35.46% to 58.09%, *P*<0.01, N = 3) and a corresponding decrease in population in G0-G1 phase (from 61.76% to 39.36%, *P<*0.01, N = 3), with the percentage of G2-M phase cells remained unchanged (Fig 4A). In contrast, when the PPIase activity was impaired, the CYPJ(R44A&F49A) mutant could no longer induce this shift in cell cycle (*P*>0.05, N = 3) (Fig 4A).

In RNAi experiments, we synthesized 3 small interference RNAs (siRNAs) specific to the coding sequence of CYPJ, and found that one of the siRNAs complex (si-J-1, 352 nt-370 nt of *CYPJ* mRNA) could decrease the expression of co-transfected exogenous CYPJ protein efficiently (Fig 4B). We further examined its effect on the expression of endogenous *CYPJ* gene in HCC cell line SK-Hep1 (Fig 4C). The siRNAs was specific for *CYPJ*, for it did not affect the expression of *CYPA* mRNA (Fig 4C). *CYPJ* knockdown resulted in a decreased population of S-phase SK-Hep1 cells (from 36.9% to 24.2%, *P*<0.01, N = 3) and increased G0-G1 phase cells (from 52.0% to 66.9%, *P*<0.01, N = 3) (Fig 4D). In addition, this result was further supported by the inhibition of CYPJ PPIase activity via CsA. After a 48 h treatment of SK-Hep1 cells with 4 μM CsA, a significant increase in the percentage of G0-G1 phase cells (from 51.7% to 60.4%, *P*<0.01, N = 3) was observed accompanied by a significant decrease in S phase cells (from 36.7% to 25.2%, *P*<0.01, N = 3) with G2-M phase unchanged (Fig 4F).

Together, these results indicated that CYPJ could promote the transition of cell cycle from G1 phase to S phase, and this function was dependent on the PPIase activity of CYPJ. This might contribute to the enhanced growth of HCC.

### CYPJ modulates cyclin D1 expression

**Fig 5:**
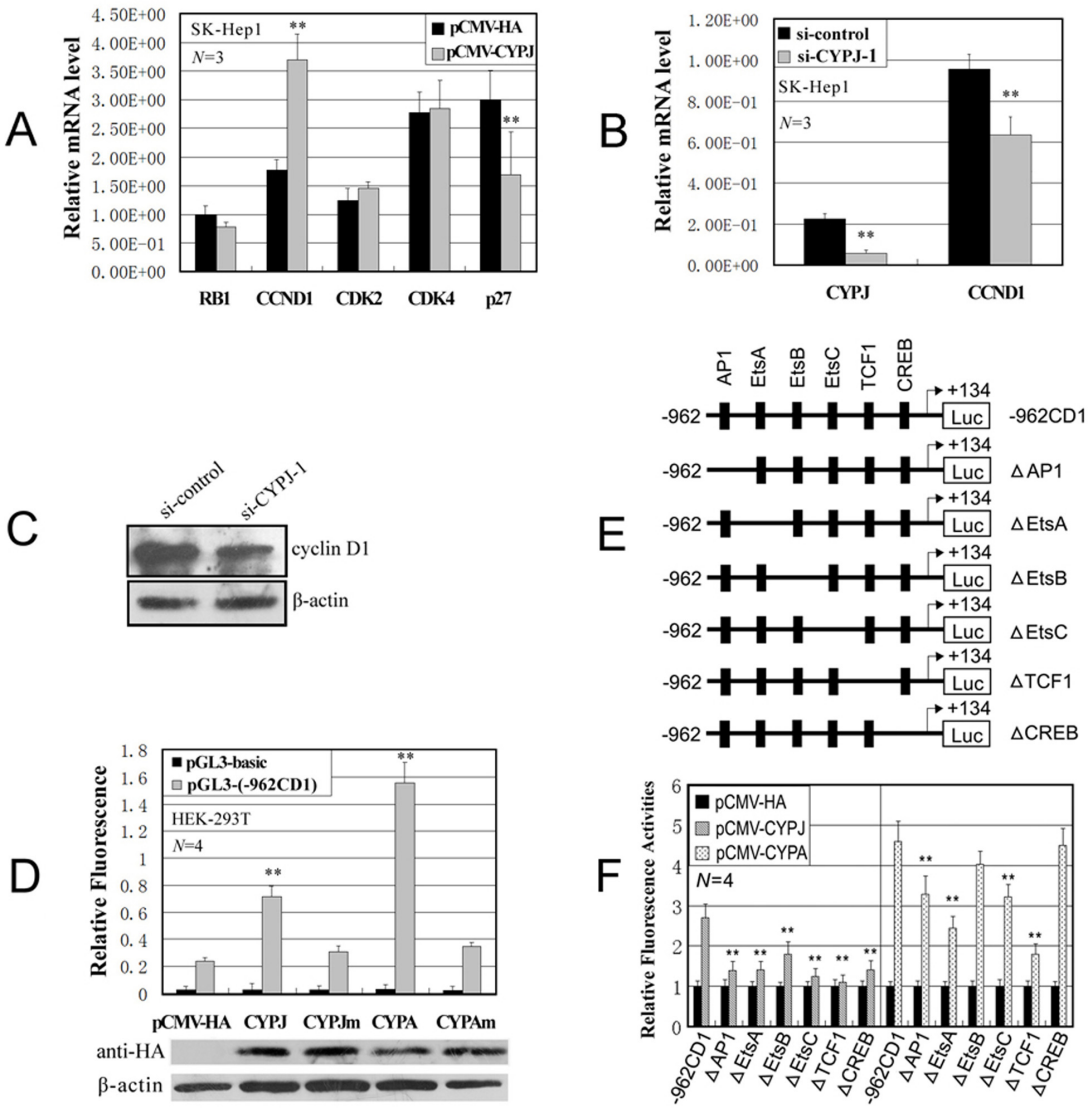
CYPJ regulates the transcription of cyclin D1. (A) Effects of CYPJ overexpression on the expression of several cell cycle controllers. The mRNA levels of each gene were evaluated by quantitative real-time RT-PCR, and subsequently normalized by internal *GAPDH*. ** *P*<0.01. (B) Knockdown of CYPJ expression decreased the expression level of cyclin D1 mRNA. ** *P*<0.01. (C) Knockdown of CYPJ expression decreased the expression level of cyclin D1 protein. (D) Regulation of cyclin D1 promoter by CYPJ, CYPA and their mutants. HEK-293T cells were cotransfected with expression vectors encoding CYPJ, CYPJ mutant (R44A&F49A), CYPA, CYPA mutant (R55A&F60A) or vector control alone (pCMV-HA) and cyclin D1 promoter reporter -962CD1 or the basic luciferase control vector (pGL3-basic) as indicated. The pRL-SV40 plasmids were used as internal control to normalize transfection efficiency. The exogenous CYPJ, CYPA and their mutants were detected by western blot using anti-HA monoclonal antibodies. ** *P*<0.01. (E) Graphics indicate the -962 cyclin D1 promoter (-962CD1) and its deletion mutants. (F) HEK-293T cells were cotransfected with vectors encoding CYPJ, CYPA or pCMV-HA vector control and cyclin D1 promoter reporter -962CD1, or various mutants. Mean and SD of relative fluorescence activities were obtained from four independent experiments. The relative fluorescence activities were normalized to control vector transfected cells. ** *P*<0.01.

Given the function of CYPJ in the G1-S transition, we next examined gene expression related to G1-S phase entry in CYPJ transfected cells by quantitative real-time RT-PCR. As shown in Fig 5A, the expression of *RB1*, cyclin D1 (*CCND1*), *CDK2*, *CDK4*, and p27 was examined. Compared with control cells, CYPJ overexpression upregulated the expression of cyclin D1 and repressed the expression of p27. In a complementary experiment, knockdown of CYPJ led to a decrease in the expression of cyclin D1 (Fig 5B and 5C).

To further evaluate the mechanism by which cyclophilins regulate the transcription of cyclin D1, we examined the effects of overexpressing CYPJ and CYPA on cyclin D1 promoter using cyclin D1-luciferase reporter constructs (-962CD1), as described previously [33]. Compared with control cells transfected with empty vector, CYPJ and CYPA overexpression stimulated the transcription of cyclin D1 promoter for about 3 folds and 7 folds, respectively (Fig 5D). Interestingly, the stimulation of the cyclin D promoter by CYPJ and CYPA is dependent on their PPIase activity, as the PPIase-deficient mutants CYPJ(R44A&F49A) and CYPA(R55A&F60A) failed to activate cyclin D1 promoter (Fig 5D). The CYPJ-mediated activation of cyclin D1 promoter was also inhibited by CsA treatment in a dosage-dependent manner (Data not shown). It is thus likely that the effect of CYPJ on cell cycle progression can be attributed at least in part to its activation of the transcription of cyclin D1.

The cyclin D1 promoter contains binding sites for several transcription factors, including AP1, EtsA, EtsB, EtsC, TCF1 and CREB (Fig 5E). To determine sites required for cyclophilin mediated cyclin D1 activation, we examined the effects of CYPJ and CYPA on a series of cyclin D1 promoter mutants with each site deleted (Fig 5F). The results indicated that, deletion of any transcription factor binding site significantly diminished the activation of cyclin D1 promoter by CYPJ (*P*<0.01), and the deletion of AP1, EtsA, EtsC or TCF1 affected the activation of cyclin D1 promoter by CYPA (*P*<0.01). These results suggested that cyclophilin signals might regulate cyclin D1 transcription through multiple ways, which differs from other proteins such as FAK [34] or SNIP1 [35] that activate cyclin D1 promoter through a unique transcription factor binding site.

### Elevated expression of CYPJ promotes HCC

**Fig 6:**
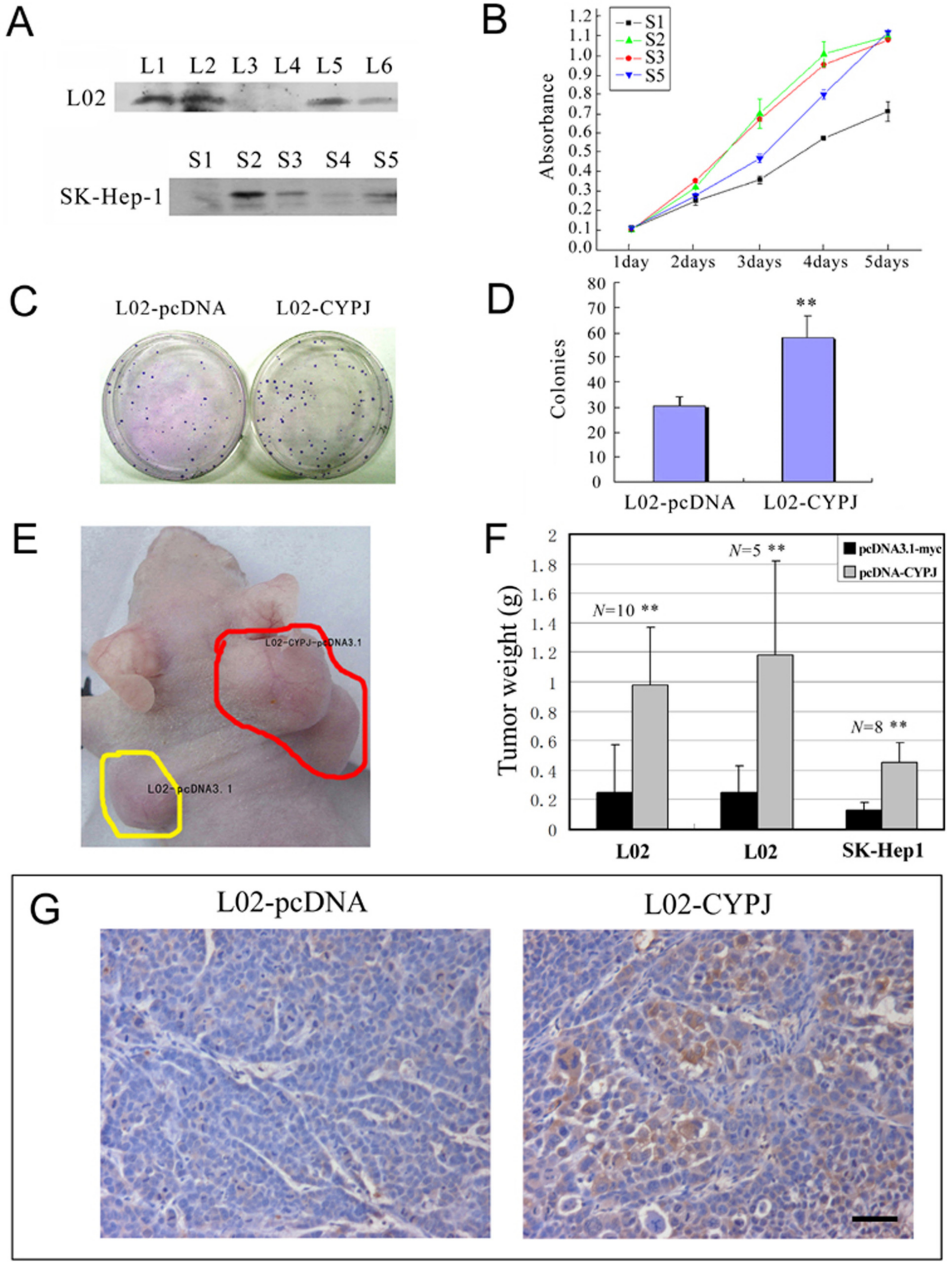
Overexpression of CYPJ promoted the growth of human cell line SK-Hep1 and L02. (A) Cell clones with CYPJ protein by stable cell transfection were identified by western blot using anti-myc monoclonal antibodies. L3, L4 and S1 are negative control cells stably transfected with empty vector pcDNA3.1-myc. (B) Growth curves of the recombinant cells with or without exogenous CYPJ were obtained from MTS assays. Each sample was tested in triplicate and the error bars are included. (C) and (D), promotion of colony formation by CYPJ in normal liver cell line L02. (C) Expression of CYPJ promoted the colony formation in L02 cells. L02 cells were transfected with either pcDNA3.1-myc vector (left) or with CYPJ-expression vector (right). (D) Percentage of G418 resistant colonies. Data were results of 3 independent experiments. ** *P*<0.01. (E), (F) and (G), CYPJ promoted *in vivo* tumorigenicity of L02 and SK-Hep1 cells. (E) Recombinant cells with or without exogenous CYPJ were injected into each side of nude mice, respectively, and tumor weight was measured. (F) Compared with control tumors, tumors originated from cells with overexpressed CYPJ were significantly heavier. ** *P*<0.01. (G) Immunohistochemical staining using anti-myc monoclonal antibody indicated the expression of exogenous CYPJ. Scale bar indicated 50 μm.

To investigate the role of CYPJ in HCC tumorigenesis, normal hepatocytes L02 cells and HCC cell line SK-Hep1 were used to generate stable cell lines expressing CYPJ, and cell growth was monitored. After transfection and selection for 3 weeks, we obtained a series of clones that stably expressed CYPJ (Fig 6A; L1, 2, 5, and 6 in L02 cell line, and S2, 3, 4, and 5 in SK-Hep1 cell line). L3, L4 and S1 clones are control cells stably transfected with empty vector. SK-Hep1 clones S2, S3, S5 and empty vector clone S1 were seeded in 96 well plates (1000 cells per well), and cell growth was examined in a 5-day MTS assay. As shown in Fig 6B, all three selected SK-Hep1 clones grew faster than control cells.

In colony formation assay, pcDNA-CYPJ plasmid and control plasmid pcDNA3.1-myc were transfected into L02 cells under selection with G418 for 3 weeks, and colony numbers were calculated. L02 cells transfected with CYPJ showed a significant increase in the number of colonies (about 2 folds, Fig 6C and 6D). These effects were reproducible and was observed in 3 independent experiments (*P*<0.01). Colonies were selected, and exogenously expressed CYPJ was validated by western blot (data not shown). These results suggested that elevated expression of CYPJ might contribute to the transformation of liver cells.

To further determine the role of CYPJ in tumorigenesis, we selected three stable clones (L02 clones L1, L2 and SK-Hep1 clone S2) with higher exogenous expression and inoculated them to nude mice (control clones L3, L4 and S1 were also used). We inoculated control cells and stable cell clones to the same nude mice: one on left shoulder and one on right shoulder. As shown in Fig 6E, the stable cell clones gave rise to much larger tumors than the vector controls. In 10–14 days, tumors emerged. Tumors were later removed at day 40 from the mice and were individually weighed. Tumors originated from the L02-CYPJ cells weighed 392% and 472% respectively, of those of the vector controls, and tumors originated from the SK-Hep1-CYPJ cells weighed 346% of the vector controls (Fig 6F). The exogenous expression of CYPJ was also detected in L02-CYPJ-originated tumors by immunohistochemistry (Fig 6G). These results indicated that CYPJ could promote liver tumor formation not only *in vitro* but also *in vivo*.

### Targeting CYPJ diminishes the growth of liver cancer cells

**Fig 7:**
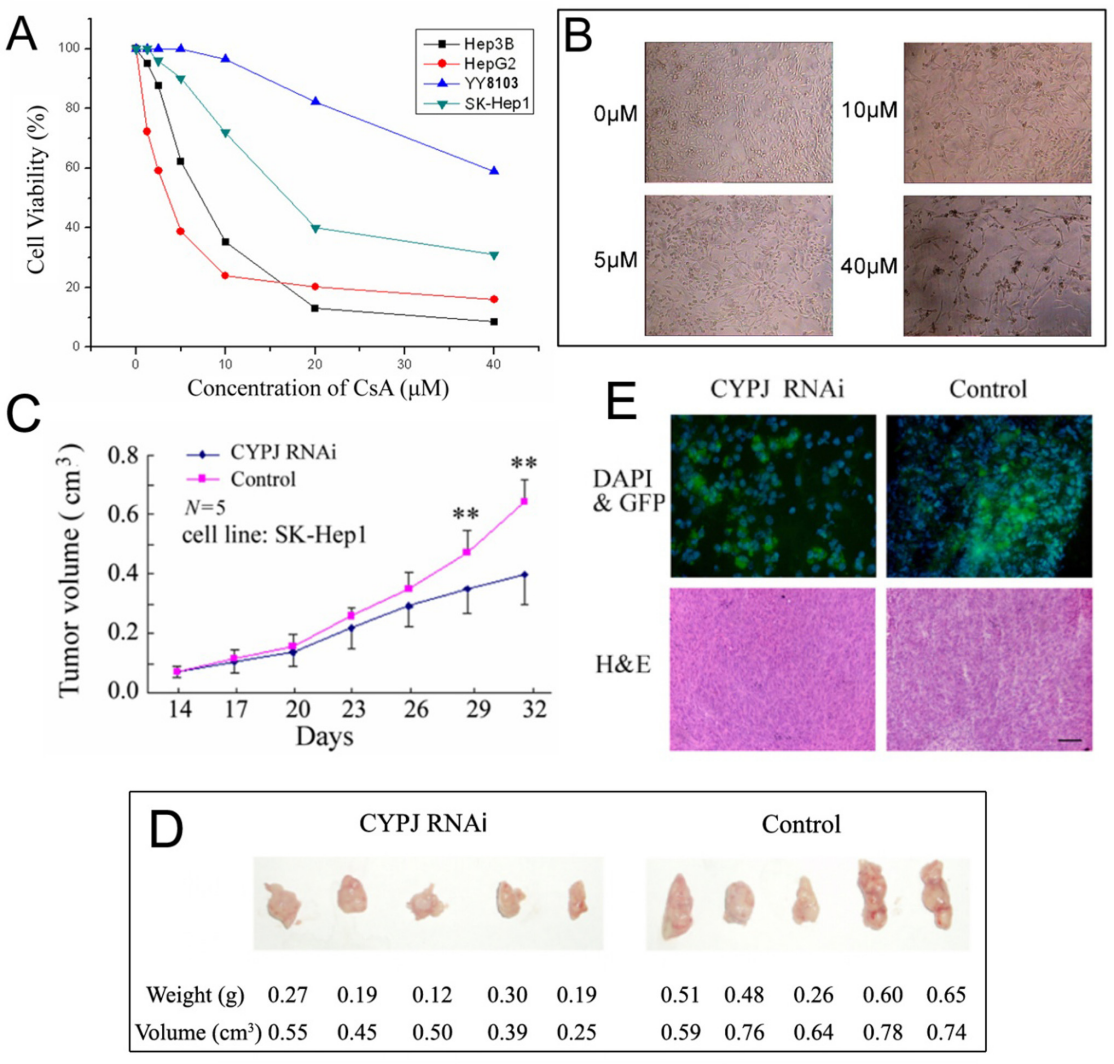
Targeting CYPJ diminishes the growth of liver cancer cells. (A) and (B), CsA inhibited the growth of several liver cancer cell lines. (A) Cell viability of liver cancer cell lines in CsA treatment. (B) Microscopic view of SK-Hep1 cells treated with different concentrations of CsA. (C), (D) and (E) Lentivirus-mediated CYPJ knockdown resulted in slower tumor growth *in vivo*. (C) Tumor growth curves of SK-Hep1 liver cancer cell line originated from tumors injected with LV-CYPJ-RNAi or LV-non-silencing control. ** *P*<0.01. (D) Comparison of the hepatic tumor weight and volume 32 days after injection of Lentivirus between CYPJ knockdown mice and control. (E) Immunofluorescence and pathological analysis of mouse liver cancer cells with/without CYPJ knockdown. For immunofluorescence, transducted cells were labeled with GFP marker and cell nucleus were labeled with DAPI. For pathological analysis, tissue sections were stained with H&E. Scale bar below indicated 100 μm.

As exogenous expression of CYPJ can promote the growth of liver cells, we examined the effects of inhibition of CYPJ on liver cancer cell growth. The CYPJ inhibitor CsA was used to inhibit the activity of CYPJ, and lentivirus mediated RNAi was used to knockdown CYPJ expression. CsA inhibited the growth of several liver cancer cells, such as Hep3B, HepG2, YY8103 and SK-Hep1 (Fig 7A and 7B). As CsA can block the activities of most cyclophilin family members including CYPJ, the extent to which the growth inhibition by CsA can be attributed to CYPJ remained to be further established.

Using the optimal siRNA CYPJ knockdown sequence previously described, we made lentivirus carrying the corresponding shRNA of CYPJ. By quantitative real-time RT-PCR, we validated the efficiency of CYPJ shRNA lentivirus to knock down the expression of *CYPJ* in the transduced SK-Hep1 cells (data not shown). We then inoculated SK-Hep1 cells to nude mice. After 5–7 days, 10 mice were selected containing tumors of nearly the same volume (about 10–30 mm^3^). We then carried out CYPJ knockdown by injecting five mice with control viruses and other five with CYPJ shRNA viruses. The injection was repeated once in two days. The size of each tumor was determined every three days thereafter (Fig 7C). The volume of CYPJ knockdown tumors was significantly smaller than the control tumors (*P*<0.05 at day 26, and *P*<0.01 at day 29 and 32). At day 32, CYPJ knockdown tumors were 40% smaller than the controls (Fig 7C and 7D). Cell nuclei were stained by DAPI, and infected cells were detected by fluorescence microscope using the GFP marker in transduced cells (Fig 7E). These results demonstrated that knockdown of CYPJ could significantly diminish the growth of liver cancer cells *in vivo*, and CYPJ may serve as a new target for liver cancer therapy.

## Discussion

In this study, we characterized CYPJ both *in vitro* and *in vivo*. We demonstrated that CYPJ was a *bona fide* PPIase that was sensitive to inhibition by CsA, albeit with low potency. We found that upregulation of *CYPJ* was related to the clinical grades of HCC. We further demonstrated that overexpression of CYPJ caused an increase in cell proliferation and knockdown of CYPJ inhibited HCC cell growth both *in vitro* and *in vivo*, thus establishing CYPJ as a potential target for developing novel anti-cancer agents.

The dominant nuclear accumulation of CYPJ is similar to some other prolyl isomerases, such as Pin1, CYPA, and FKBP25. Previous studies have shown that CYPA could bind to YY1 and alter its transcriptional activity [36]. Pin1, a PPIase specific for phosphotyrosyl-prolyl residues, can interact with phosphorylated c-Jun, and dramatically increases its ability to activate the cyclin D1 promoter [37]. In addition, Pin1 has also been shown to interact with and activate p53 upon DNA damage [38, 39]. FKBP25 was suggested to be recruited to preribosomes to chaperone one of the protein components of the ribosome large subunit [40]. It is a regulator of the p53 pathway, which induces the degradation of MDM2 and activation of p53 pathway [41].

In this study, we found that CYPJ promoted cell cycle transition from G1 to S phase in a PPIase-dependent manner through the upregulation of cyclin D1. This was in accordance with the report that CsA, the CYPJ inhibitor, inhibits colon carcinoma cell growth by delaying cell cycle progression and induction of necroptosis [42]. Interestingly, both CYPA and CYPJ were capable of upregulating the expression of cyclin D1 in a PPIase-dependent manner, suggesting that these two members of cyclophilin family are functionally redundant for the positive regulation of Cyclin D1 expression. That CYPJ-induced transcription of cyclin D1 was dependent on multiple transcription factors suggested that it is likely to activate an early step of a physiological signaling pathway that is integrated in the *CCND1* promoter.

Using the stable SK-Hep1 and L02 cell lines, we found that increased CYPJ expression accelerated tumor cell growth *in vitro* and *in vivo*. And knockdown of CYPJ expression by lentivirus-meidated siRNA in SK-Hep1 cells significantly diminished tumor growth *in vivo*. In addition, it was reported that the CYPJ inhibitor CsA could inhibit the growth of human transitional cell carcinoma of bladder cell lines EJ cells *in vivo* [43]. Another study also found that tumor size was significantly reduced in a mouse B-lymphoid tumor model following CsA treatments [44]. These results suggested that expression of CYPJ is necessary and sufficient to support tumor growth. It has been previously reported that CYPA is upregulated in several tumor types including non-small cell lung cancer, pancreatic cancer, breast cancer, colorectal cancer, squamous cell carcinoma and melanoma [45]. CYPA promotes the growth of non-small cell lung cancer *in vivo* [46] while downregulation of cyclophilin A by siRNA diminishes non-small cell lung cancer cell growth and metastasis [47]. PPIL1 has also been reported to be upregulated in colon cancer tissues, and capable of promoting the growth of colon cancer cells through SNW1/SKIP and/or stathmin [29]. Cyclophilin D is a component of permeability transition-pore, which was found to be overexpressed in breast cancer tissues, acting as a suppressor of apoptosis [48]. These findings suggested that promotion of tumor growth may be a shared property of members of the cyclophilin family.

Our results clearly demonstrated that inhibitors of CYPJ are of benefit in HCC therapy. CsA, an immunosuppressive drug, is a potent inhibitor of CYPA. CsA has been used in liver cancer cells in combination with taxol to overcome drug resistance [49]. However, CsA has drawbacks as cancer therapeutic agent, due to its immunosuppressive activity [50]. Non-immunosuppressive CsA derivatives and novel inhibitors designed based on the structure of CYPJ and CYPJ/CsA complex may lead to improvement to clinical outcome for patients with HCC.

## Conclusions

Our study demonstrated that expression of the PPIase CYPJ is likely to facilitate tumor growth by promoting cell cycle transition from G1 to S phase in a PPIase-dependent manner through the upregulation of cyclin D1. This enables CYPJ to be a promising therapeutic target for hepatocellular carcinoma and for developing novel anti-cancer agents.

## Supporting Information

S1 Fig 3D-Structures of human CYPJ. (A) Ribbon diagram of CYPJ showing the active site pocket. Helices and β-strands were shown in green and blue, respectively. Disulfide bridges were shown in pink. The N and C termini were labeled. Side chains of His43, Arg44 and Gln52 were shown in red. (B) Side chains of His43, Arg44 and Gln52 of CYPJ in structures refined at 2.0 Å resolution and at 2.1 Å resolution (using two different X-ray data sets) were shown in red and green, respectively. Those in the reported structures of the unligated CYPA, the CYPJ-CsA complex, as well as CYPA in complexes with dipeptide AP and tetrapeptide AAPF were also superimposed with those in CYPJ, which were shown in light blue, yellow, dark blue and pink, respectively.
(TIF)

S1 Table Crystal and statistical data and crystallographic refinement. (DOCX)

S2 Table Primer sequences for quantitative real-time RT-PCR. (DOCX)

S3 Table Hydrogen bonding interactions between CYPJ and CsA^*a*^. (DOCX)
