# Supplementary figures and images for: Cyclophilin J Is a Novel Peptidyl-Prolyl Isomerase and Target for Repressing the Growth of Hepatocellular Carcinoma
Source: PLoS One. 2015 May 28;10(5):e0127668. doi: 10.1371/journal.pone.0127668 (PMC4447340; doi:10.1371/journal.pone.0127668)

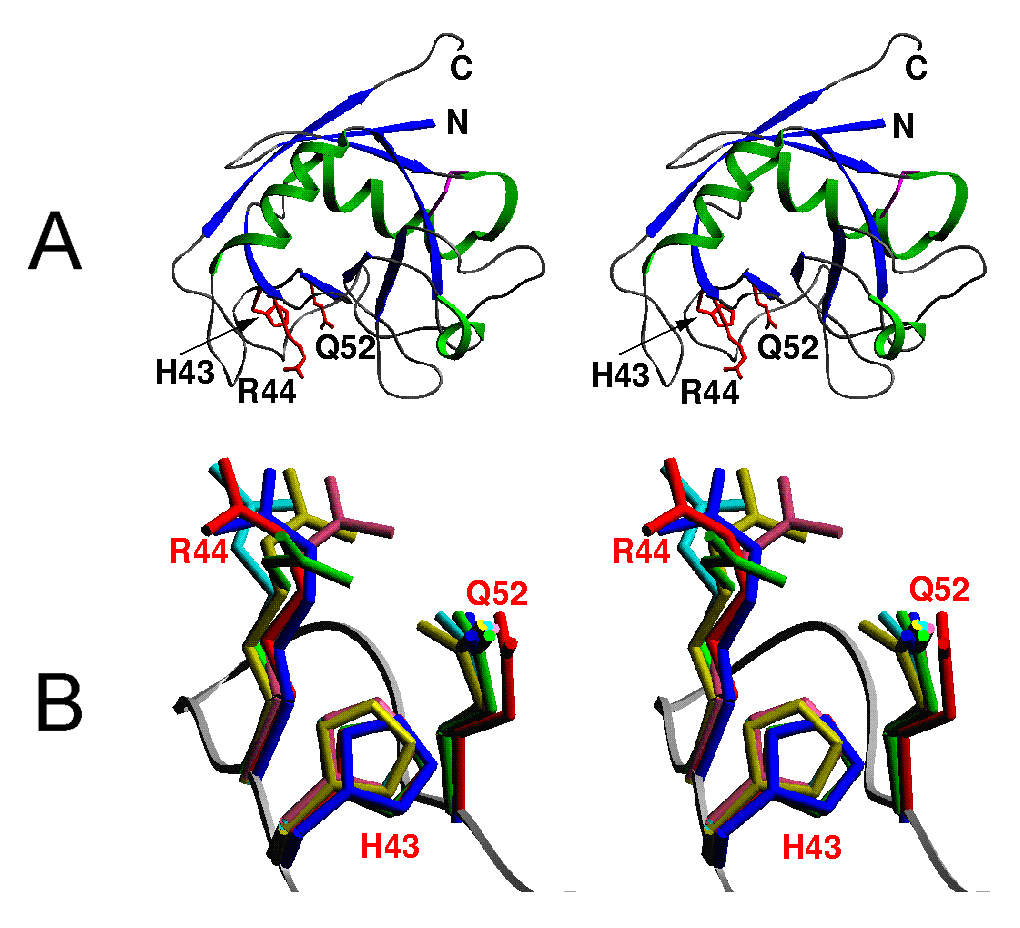

Supplement: S1 Fig — (A) Ribbon diagram of CYPJ showing the active site pocket. Helices and β-strands were shown in green and blue, respectively. Disulfide bridges were shown in pink. The N and C termini were labeled. Side chains of His43, Arg44 and Gln52 were shown in red. (B) Side chains of His43, Arg44 and Gln52 of CYPJ in structures refined at 2.0 Å resolution and at 2.1 Å resolution (using two different X-ray data sets) were shown in red and green, respectively. Those in the reported structures of the unligated CYPA, the CYPJ-CsA complex, as well as CYPA in complexes with dipeptide AP and tetrapeptide AAPF were also superimposed with those in CYPJ, which were shown in light blue, yellow, dark blue and pink, respectively. (TIF) [file pone.0127668.s001.tif]
