# Supplementary material for: Cyclophilin J Is a Novel Peptidyl-Prolyl Isomerase and Target for Repressing the Growth of Hepatocellular Carcinoma
Source: PLoS One. 2015 May 28;10(5):e0127668. doi: 10.1371/journal.pone.0127668 (PMC4447340; doi:10.1371/journal.pone.0127668)
Supplement: S1 Table — (DOCX) [file pone.0127668.s002.docx]

**Table S1. Crystal and statistical data and crystallographic refinement.**

|  | | | CYPJ | CYPJ-CsA complex |
| --- | --- | --- | --- | --- |
|  |  |  |  | (data set 2) |
| Space group | | | P3_1_21 | P3_1_21 |
| Unit-cell parameter (Å) | | a, b | 40.6 | 64.4 |
|  |  | c | 170.7 | 200.9 |
| No. of molecule per asymmetric unit | | | 1 | 2 |
| Resolution (Å) | | | 2 | 2.4 |
| No. of unique reflections | | | 11753 | 19272 |
| Data completeness (%) | | | 99.4（99.7）^a^ | 97.6 (83.4) ^a^ |
| R_merge_ (%) ^d^ | | | 3.6（18.8）^a^ | 7.6 (40.6) ^a^ |
| Reflections with I >3σ(I) (%) | | | 92.7（82.4）^a^ | 72.1 (32.1) ^a^ |
| No. of amino acid residues | | | 160 | 159 / 160 *^b^* |
| No. of CsA molecule | | | 0 | 2 |
| No. of solvent molecules | | | 162 | 32 |
| No. of Ni^++^ | | | 2 | 0 |
| R-factor (%) *^c^* | | | 20.2 (22.2) ^a^ | 19.3 (35.9) ^a^ |
| R _free_ (%) | | | 25.4 (29.3) ^a^ | 23.7 (43.0) ^a^ |
| Rmsd *^d^* | Bond length (Å) | | 0.0051 | 0.0065 |
|  | Bond angle (º) | | 1.2 | 1.3 |
|  | Main chain | | 29 | 51 |
| Mean temperature | Side chain | | 31 | 53 |
| Factors (Å^2^) | Solvent | | 37 | 42 |
|  | CsA | |  | 42 |
| *^a^* Numbers in parentheses correspond to the data in the highest resolution shell, 2.07-2.00 Å for CYPJ and 2.49-2.40 Å for CYPJ-CsA, respectively. | | | | |
| *^b^* Numbers of the amino acids for molecules A/B in an asymmetric unit. | | | | |
| *^c^* R_merge_=SUM [ ABS (I - <I> ) ] / SUM (I) | | | | |
| *^d^* Root-mean-square-deviation | | | | |
