# Supplementary material for: Cyclophilin J Is a Novel Peptidyl-Prolyl Isomerase and Target for Repressing the Growth of Hepatocellular Carcinoma
Source: PLoS One. 2015 May 28;10(5):e0127668. doi: 10.1371/journal.pone.0127668 (PMC4447340; doi:10.1371/journal.pone.0127668)
Supplement: S2 Table — (DOCX) [file pone.0127668.s003.docx]

**Table S2. Primer sequences for quantitative real-time RT-PCR.**

| Gene name | Forward primer (5’-3’) | Reverse primer (5’-3’) |
| --- | --- | --- |
| GAPDH | CACTCCTCCACCTTTGACG | ACCACCCTGTTGCTGTAGC |
| CYPA_CDS | AACTTCATCCTAAAGCATACGG | TTGCCATCCAACCACTCAG |
| CYPJ_CDS | CATCACCTATGGCAAACAGC | TGGCAACTTCTCCAACTCATC |
| CCND1 | GAACACGGCTCACGCTTACCTC | ACTTGTGCCCTTGCCCCATC |
| RB1 | CAGCAGAAACTGGCAGAAATGAC | TGTCCACCAAGGTCCTGAGATC |
| CCNE1 | GCGTCGCTGATGAAGATGC | GAGAGGAGAAGCCCTATTTTG |
| CCNA2 | AGTATTTGCCATCAGTTATTGCTGG | GGTAGGTCTGGTGAAGGTCC |
| CDK2 | GGATGAAGATGGACGGAGC | CCTGGAAGAAAGGGTGAGC |
| CDK4 | TGACTGGCCTCGAGATGTATC | CAGAGATTCGCTTGTGTGGG |
| P27 | AAAATGTTTCAGACGGTTCCC | CCTTGCTTCATCAAGCAGTG |
| P16 | AACCATGCCCGCATAGATG | GTAGGACCTTCGGTGACTGATG |
