# Supplementary material for: Cyclophilin J Is a Novel Peptidyl-Prolyl Isomerase and Target for Repressing the Growth of Hepatocellular Carcinoma
Source: PLoS One. 2015 May 28;10(5):e0127668. doi: 10.1371/journal.pone.0127668 (PMC4447340; doi:10.1371/journal.pone.0127668)
Supplement: S3 Table — (DOCX) [file pone.0127668.s004.docx]

**Table S3. Hydrogen bonding interactions between CYPJ and CsA*^a^*.**

| Atom in CsA | Atom in hCyPJ | Distance（Å） |
| --- | --- | --- |
| MeBmt1 O | Gln52 NE2 | 3 |
| MeBmt1 CA | Asn91 O | 3.2 |
| MeBmt1 CD2 | Asn91 O | 3.1 |
| Abu2 O | Asn92 ND2 | 2.8 |
| Abu2 N | Asn92 OD1 | 2.8 |
| Sar3 N | Gly60 O | 3.2 |
| Sar3 CN | Gly60 O | 3.1 |
| MeLeu4 CN | Asn92 OD1 | 3.2 |
| MeLeu9 O | His110 NE2 | 3 |
| MeLeu9 O | Phe49 CZ | 3.2 |
| MeLeu10 O | Arg44 NH1 | 3.1 |
| MeLeu10 O | Arg44 NH2 | 3.3 |
| MeVal11 CN | Tyr115 OH | 2.9 |
| *^a^* Average values for molecules A and B. | | |
